# Japanese Encephalitis among Patients with Acute Encephalitic Syndrome Admitted to a Tertiary Hospital in Chitwan, Nepal – A Prospective Observational Study

**DOI:** 10.1371/journal.pone.0099999

**Published:** 2014-06-20

**Authors:** Sundar Twayana Ram, Ami Neuberger, Lekh Jung Thapa, Rana Pramendra Vir Singh, Ben Shofty, Eli Schwartz

**Affiliations:** 1 Faculty of Internal Medicine, Kathmandu University Hospital, Dhulikhel, Nepal; 2 Unit of Infectious Diseases & Internal Medicine B, Rambam Medical Center, Bruce Rappaport Faculty of Medicine, Technion Institute of Technology, Haifa, Israel; 3 Department of Neurology, College of Medical Sciences, Bharatpur, Nepal; 4 Departments of Neurology and Medicine, College of Medical Sciences, Bhartapur, Nepal; 5 The Gilbert Israeli Neurofibromatosis Center, Tel Aviv Medical Center, Tel Aviv, Israel; 6 The Center for Geographic Medicine and Tropical Diseases, Sheba Medical Center & Sackler Faculty of Medicine, Tel Aviv University, Tel Aviv, Israel; Fondazione IRCCS Policlinico San Matteo, Italy

## Abstract

**Introduction:**

The reported incidence of JE among patients with acute encephalitic syndrome (AES) in Nepal ranges between 20% to 62%. In light of the lack of up-to-date data, we sought to describe the epidemiology of JE in Chitwan, Nepal.

**Methods:**

A prospective observational study was conducted during 2010–2012 in the College of Medical Science in the Chitwan District. Patients with suspected JE were tested for anti-JE IgM in serum and cerebrospinal fluid (CSF).

**Results:**

Of 227 all patients tested, 18 (7.9%) were found positive for JE. 17/202 (8.4%) patients with AES had JE. All, with the exception of two patients, were diagnosed on the basis of positive a serologic test, both in serum and CSF samples. Patients with JE were significantly older (42.1±27.6 years) than patients without JE (25.6±25.2 years, p = 0.02). Half of JE cases occurred in adults older than 50. More of the JE cases (11/18, 61.1%) occurred during the rainy season when compared to the JE negative patients [71/209, (34%), p = 0.01]. None of the JE patients had a relevant travel history, and one recalled having been immunized against JE. There was a variation in the geographic distribution of cases across the districts of the central Terai.

**Conclusions:**

In this cohort, the proportion of patients with AES who had JE was lower than in previous studies. In addition, most patients were adults, and cases were not distributed uniformly across the central Terai region. The risk of acquiring JE by short-term travelers in the area is likely to be low. Vector-control programs and the promotion of mosquito avoidance behavior in the Terai region should continue. The high proportions of adults among patients with JE may suggest recent changes in the epidemiology of JE in the central Terai region, and routine immunization of all adults should be considered.

## Introduction

Japanese encephalitis (JE) is a common cause of acute encephalitic syndrome (AES) in Southeast Asia. The disease is caused by the JE virus, and is transmitted from animal host to humans through a mosquito vector. The clinical manifestations of JE range from asymptomatic infections to devastating encephalitis syndrome associated with appreciable mortality and frequent central nervous system (CNS) sequelae in survivors [1].

**Figure 1:**
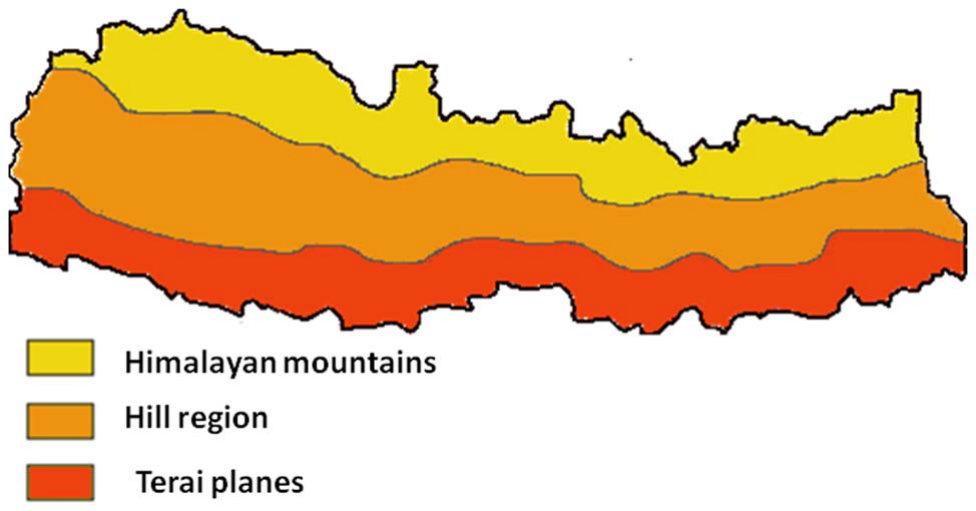
Geographical zones of Nepal.

Since the first report of JE in Nepal in 1978, more than 30,000 cases have been reported in the country, usually occurring from June to November, during the rainy season and the post-monsoon period [2]–[3]. A comprehensive, hospital-based JE surveillance was performed in Nepal in 2004–2006, showed that that showed that the majority of laboratory-confirmed cases are found in the 24 districts of the low-lying Terai plains bordering India, with additional cases found sporadically or in small outbreaks in other, more elevated regions of the country, including the Kathmandu valley [2], [4]–[6] (see figure 1). Within the Terai region, JE incidence and mortality rate are higher in four hyperendemic western districts - Kailali, Bardiya, Banke, and Dang [2]. The Chitwan district, located in the central Terai, has a population of 579,984, and is visited by thousands of tourists every year. In the past, fewer cases were reported in Chitwan district than in the hyperendemic districts and in any other neighboring district in the central Terai [2].

The reported incidence of JE among Nepalese patients with AES is around 25%, but ranges between 20% to 62% [6]–[8]. This wide variation can be explained in part by different disease incidence rates in various geographic areas, by marked changes in disease incidence over time, and by inhomogeneous modes of JE diagnosis used. In most previous studies, JE was diagnosed with the use of a single serum IgM antibody measurement. Serum IgM studies may be falsely negative early in the course of the disease, and falsely positive when cross-reacting with other flaviviruses, which are also found in Nepal (e.g. the dengue fever virus and probably also the West Nile virus) [9]–[10]. Since most JE infections are asymptomatic, the World Health Organization (WHO) recommend testing of cerebrospinal fluid (CSF) in endemic countries whenever feasible, in order to avoid wrongly implicating asymptomatic JE as the cause of AES [11].

In light of the changing epidemiology of JE in Nepal, the introduction of JE vaccination in endemic areas, the reliance on a single serum IgM anti-JE antibody measurement in most previous studies, and the lack of up-to-date published data, we sought to describe the epidemiology and clinical characteristics of patients with JE diagnosed in a tertiary hospital which provides medical care for Chitwan and its neighboring districts. In order to improve the diagnostic accuracy we used IgM anti-JE antibodies in serum and CSF. These data are relevant both to the population of the central Terai region, and to the many tourists who frequent the area.

## Methods

This prospective observational study was conducted between January 1, 2010 and December 31, 2012 at the College of Medical Science (COMS) in Bharatpur, Chitwan District, Nepal. COMS is a 700-beds referral hospital for the population of Chitwan and the neighboring districts.

All patients admitted to the hospital with AES or with a presumed CNS infection that did not fulfill the WHO criteria for AES were included in the study. AES was defined on the basis of the WHO criteria as a clinical syndrome that included an acute onset of fever, and either a change in mental status or a new onset of seizures (excluding simple febrile seizures) [12]. Patients were defined as having a presumed CNS infection, which did not meet the WHO criteria for AES, if they were judged by the attending clinician to have an acute CNS infection, but had no fever upon admission, or had fever and meningeal irritation signs with no change in the level of consciousness and no convulsions.

Patients with AES and patients with a suspected CNS infection that did not meet the formal criteria for AES were tested for anti- JE IgM in serum and CSF samples. Both a peripheral blood smear and a rapid antigen detection kit were used to diagnose malaria. Epidemiologic and clinical data were prospectively recorded on the basis of the patients' medical charts. Epidemiologic data included gender, age, ethnic group, place of residence, travel history, and JE vaccination history. Clinical data included fever, headache, nuchal rigidity, convulsions, and decreased level of consciousness. Outcome was determined upon discharge.

Laboratory diagnosis of JE was performed in the Nepal Public Health Laboratory, Kathmandu. IgM antibody capture enzyme-linked immunoassay was used to identify anti-JE IgM in serum and/or CSF. Five mL of serum and 2 mL of CSF were used. Samples that were tested within seven days were kept refrigerated at 4°C until tested. Samples were frozen at −20°C or below if tested after more than seven days. Transportation of samples to the diagnostic laboratory in Kathmandu was performed with maintenance of the cold chain. Test kits were provided by the Indian National Institute of Virology (Pune, India). These kits have been reported to have a sensitivity of 75% and 71% in CSF and serum, and to have a specificity of 96% and 77% in CSF and serum, respectively [13].

The study was approved by the Ethics and Research Committee of the College of Medical Sciences, Bhartapur, Nepal. The committee waived the need for a written informed consent as samples were sent for analysis as part of the clinical evaluation of a patient with encephalitis. No new intervention (therapeutic or diagnostic) was performed.

## Results

A total of 227 patients underwent laboratory evaluation for JE. Of them, 202 were consecutive patients with clinical criteria of AES, and 25 additional patients had presumed CNS infection that did not meet the WHO criteria for AES. Of patients with AES, 17/202 (8.4%) were diagnosed as having JE, whereas 1/25 (4%) of the patients with a suspected CNS infection that did not fulfill the WHO criteria for AES also had JE. 225 out of 227 patients were tested for the presence of *Plasmodium* parasites; one patient with AES actually had cerebral malaria.

Altogether 18 patients were diagnosed with JE. Demographic and clinical data of these patients are presented in Table 1. All, with the exception of two patients, were diagnosed on the basis of positive anti-JE IgM antibody, both in serum and CSF samples. Two patients with JE had only a single specimen available for evaluation; one had a positive serum sample and the other a positive CSF sample.

**Table 1:** Demographic and clinical manifestations of patients with Japanese encephalitis.

	Age	Gender	District	Ethnic group	Recalled previous JE vaccination	Date of diagnosis	Anti JE antibodies in:	Clinical manifestations
**1**	30	Female	Chitwan	Newar	yes	1/2010	Serum only	AES&
**2**	2 months	Male	Makwanpur	other	no	3/2010	Serum+CSF#	AES
**3**	59	Male	Makwanpur	others	no	7/2010	Serum+CSF	AES
**4**	25	Male	Bara	others	no	1/2010	Serum+CSF	AES
**5**	63	Male	Chitwan	Rai	no	1/2011	Serum+CSF	AES
**6**	61	Male	Chitwan	Brahmin	no	2/2011	Serum+CSF	Fever, headache
**7**	20	Female	Chitwan	Kumal	no	7/2011	Serum+CSF	AES
**8**	4	Male	Chitwan	others	no	7/2011	Serum+CSF	AES
**9**	74	Male	Parsa	Brahmin	no	11/2011	Serum+CSF	AES
**10**	70	Male	Nawalparasi	Brahmin	no	6/2011	Serum+CSF	AES
**11**	6	Female	Nawalparasi	others	no	7/2011	Serum+CSF	AES
**12**	80	Female	Nawalparasi	Brahmin	no	8/2011	Serum+CSF	AES
**13**	60	Female	Nawalparasi	Brahmin	no	8/2011	Serum+CSF	AES
**14**	4	Female	Nawalparasi	others	no	8/2011	Serum+CSF	AES
**15**	72	Male	Nawalparasi	Chetri	no	8/2011	Serum+CSF	AES
**16**	37	Female	Nawalparasi	Chepang	no	8/2011	Serum+CSF	AES
**17**	62	Female	Nawalparasi	others	no	8/2011	Serum+CSF	Fever, headache, nuchal rigidity
**18**	31	Female	Rupandehi	Gurung	no	9/2011	CSF only	AES

& AES, acute encephalitis syndrome;

# CSF, cerebrospinal fluid;

A comparison between the demographic and clinical characteristics of patients with and without JE is summarized in Table 2. In the entire cohort, patients with JE were significantly older (42.1±27.6 years, median 48 years, range from two months to 74 years) than patients without JE (25.6±25.2 years, median 17 years, range from one month to 85 years, p = 0.02). Results were similar when we included only patients with AES. Only 4/18 (22.2%) of the patients with JE were younger than 18, and 9/18 (50%) of JE cases occurred in adults older than 50. The proportion of patients younger than 18 was higher among patients without JE (107/209, 51.2%, p = 0.04).

**Table 2:** Demographic and clinical characteristics of patients with and without Japanese Encephalitis.

	JEŧ positive (N = 18)	JE negative (N = 209)	P value
Age (years±SDŦ)	42.1±27.6	25.6±25.2	0.02
Patients <18 year old	4/18 (22.2%)	107/209 (51.2%)	0.04
Patients >50 year old	9/18 (50%)	44/209 (21.1%)	0.01
Gender (% female)	9/18 (50%)	80/209 (38.3%)	NS
Occuring during the rainy season (July–September)	11/18 (61.1%)	71/209 (34)%	0.01
District of residence			0.06
Chitwan	5/18 (27.8%)	98/209 (46.9%)	
Nawalparasi	8/18 (44.4%)	31/209 (14.8%)	
Makwanpur	2/18 (11.1%)	53/209 (25.4%)	
Bara	1/18 (5.6%)	9/209 (4.3%)	
Parsa	1/18 (5.6%)	3/209 (1.4%)	
Rupandehi	1/18 (5.6%)	7/209 (3.3%)	
Other	0/18 (0%)	8/209 (3.8%)	
Outcome			NS
Cured/improved	18/18 (100%)	194/209 (92.8%)	
Died	0 (0%)	2/209 (0.9%)	
Left against medical advice	0 (0%)	7/209 (3.3%)	
Transferred to a tertiary care center	0 (0%)	6/209 (2.9%)	

Ŧ SD, standard deviation;

ŧ JE, Japanese encephalitis, NS, non significant.

Of the 18 patients diagnosed with JE, nine (50%) were female. The proportion of females among patients who did not have JE was 80/209 (38.3%), p = 0.33 (Table 2).

Most JE cases (11/18, 61.1%) occurred during the rainy monsoon season, between July and September. The proportion of cases diagnosed during the same months in the JE-negative patients was lower [71/209, (34%), p = 0.01]. Nonetheless, it is noteworthy that nearly 40% of JE cases did not occur during the rainy season. Only 10 patients of the entire cohort recalled having been immunized against JE: one patient with JE and nine patients without JE. Vaccination records were not available.

All patients with JE reported that they had not travelled outside of their districts during the 2 months preceding the onset of clinical infection. Similarly, 99% of JE-negative patients did not travel outside their district of residence before becoming ill.

Eight patients with JE were from the Nawalparasi district, five from Chitwan district, and two from the Makwanpur district. One single JE case originated from each of the Bara, Parsa and Rupandehi districts (Figure 1). Patients from the Nawalparasi district accounted for 39/227 (17.2%) of all patients, and 8/18 (44.4%) of patients with JE. The difference in the distribution of AES patients with and without JE in the various districts of the central Terai region showed a trend towards significance (p = 0.06). All cases from the Nawalparasi district were diagnosed during the rainy season of 2011. We found no association between belonging to a specific ethnic group (e.g. Chetri, Newar, Brahmin, Gurung etc.) and the likelihood of having or not having contracted JE (data not shown).

Out of 227 patients, 212 were either cured or markedly improved, six patients were referred to tertiary institutions, seven patients left the hospital against medical advice, and two died. All patients with JE recuperated completely or partly. Follow-up for patients who were referred to other hospitals or who left the hospital against medical advice was not available.

## Discussion

JE continues to be endemic in the Terai region of Nepal. Although JE incidence in the central Terai region is lower than in the hyperendemic western Terai districts, the disease was not uncommon there either, and it has quite certainly been underreported. In this report we describe the epidemiological and clinical data of patients with JE diagnosed in the referral center of the Chitwan district.

The incidence of JE in Nepal appears to have decreased in recent years, probably as a result of an immunization campaign with the live attenuated JE vaccine (SA 14-14-2) [9]. The vaccine was first administered in certain high-risk districts of the Terai region, and was later included in the routine childhood immunization schedule in endemic areas of the country [9]. As a policy, adults are routinely immunized with the SA 14-14-2 vaccine only in high-risk districts, despite the fact that a relatively large proportion of JE cases (38–48% in the most comprehensive survey) in all infected regions actually occur in adults [2], [14]–[15].

In this cohort, the proportion of patients with AES who had JE was lower than in past studies; 8.4% in the current study versus 20–62% in previous studies [2]–[3], [6]–[7]. This observation may reflect decreasing incidence of JE in the central Terai region due to increasing vaccination coverage. An increasing proportion of AES cases caused by another pathogen (e.g. dengue virus) is also possible theoretically. It is, however, impossible to make any definite statement about JE incidence trends since the various studies published thus far have used different laboratory diagnostic tests and were performed in different settings and geographic locations.

Current recommendations by the WHO define a probable JE case as one that “occurs in close geographic and temporal relationship to a laboratory-confirmed case of JE, in the context of an outbreak” [11]. AES, however, can be associated with other viruses, bacteria, parasites and non-infectious causes. Our results suggest that the proportion of JE cases among patients with AES is decreasing, and that the epidemiology of JE is quite different than that of AES not associated with JE. Therefore, the straightforward use of AES as a “surrogate marker” for JE may no longer be appropriate.

Patients with JE in our cohort were older than patients without JE, and most patients with JE were adults. Half of patients with JE were actually older than 50 - an unexpected observation which deserves attention when one considers the fact that JE in endemic areas occurs mainly among children. A comprehensive survey performed about a decade ago in Nepal has shown that the majority (62%) of cases in the non-Western Terai district occurred in children, and only 13% occurred in patients older than 35 [2]. The authors of the above-mentioned survey have suggested that cases may occur in adults who migrated to the Terai region from other, non-endemic, regions of the country. It is also possible that immunizations are now providing protection to a growing cohort of vaccinated children, whereas the unvaccinated adults continue to be susceptible to the disease. The introduction of JE to previously unaffected areas of the Terai would also explain the high proportion of infected adults. The fact that all cases from the Nawalparasi district occurred during the same season may suggest that this is indeed occurring. Lastly, since the incidence of the disease in the Central Terai region is not as high as in the western districts, it is possible that not all children are exposed to JE during their first years of life, and therefore more cases occur among adults [2], [9], [14]–[15]. Our findings suggest that introducing routine immunization for adults should be considered for the Central Terai region. The availability of newer vaccines with a more convenient schedule can make this goal more realistic.

The geographic distribution of cases is somewhat unexpected. People from the Chitwan district itself account for most patients admitted to COMS. However, during the study period more cases came from the Nawalparasi district (with a population of 643,508, compared with 579,984 in the Chitwan district), and less than a third of all JE cases came from the Chitwan district itself. Therefore, our data may reflect a lower incidence of JE in the Chitwan district itself when compared to some neighboring districts. This observation is supported by data gathered in a previous survey [2].

**Figure 2:**
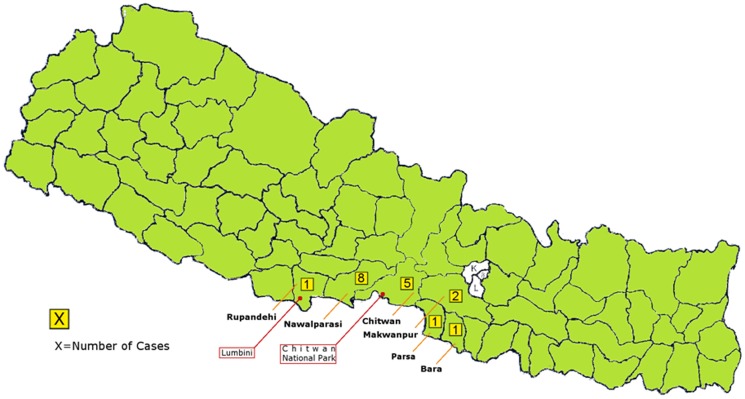
18 cases of Japanese Encephalitis diagnosed in Chitwan as per patients' district of residence.

Thousands of foreign tourists visit the Chitwan national park yearly, and others go to Lumbini, Buddha's birthplace, which is located in the neighboring Rupandehi district (Figure 2). Current recommendations do not include routine vaccination for short-term visitors to the area, and the available data from this study do not support a change to this recommendation for a number of reasons: the incidence of JE in the Chitwan district is lower than in the western Terai districts; no tourists presented to COMS with AES during the study period, and to the best of our knowledge tourist in Nepal has ever contracted JE in the country [16]–[17]. It would be wrong, however, to state with certainty that the risk of JE to travelers is decreasing. It may well be that the risk for the Nepalese population is decreasing because of increasing vaccine coverage. Since JE is a zoonotic disease, the observed decrease in JE among the Nepalese may not be relevant for the unimmunized traveler, unless vector control measures have also contributed to a decline in JE incidence. The occurrence of JE mainly among adults in the central Terai may also suggest, as discussed above, that JE is introduced into new areas in the region.

This study has several limitations. Some underestimation of JE is possible, although the combined sensitivity of CSF and serum antibodies (each more than 70% sensitive by itself) is likely to be high. The use of JE virus PCR may be appropriate in future studies [18]. False positive results caused by other flaviviruses are theoretically possible, but less likely in our report since both serum and CSF samples were positive for nearly all patients with JE, and since the reported specificity of CSF samples is over 96%. Follow-up of patients who were referred or who left the hospital against medical advice was not available. In Nepal, it is not uncommon for patients who are deemed to have a very bad prognosis to be taken by their relatives to die at home. It is also likely that patients that were referred had more profound CNS depression, and may have required intensive care. It thus follows that the mortality rate in this cohort was probably underestimated. Additional limitations of the study include its single center design and the relatively small number of patients with AES and JE.

## Conclusions

JE continues to be endemic in the central Terai district, and is likely to be underdiagnosed and underreported. The proportion of patients with AES who have JE was lower in this cohort of patients during 2010–2012, than in any other, earlier reports. Whether this difference reflects a true decrease in JE incidence cannot be ascertained by the currently available data. The high proportions of adults among patients with JE may suggest recent changes in the epidemiology of JE in the central Terai region. Our data suggest that JE incidence varies within the central Terai region, with relatively fewer cases among patients from the Chitwan district itself. All in all, JE risk to travelers visiting the central Terai is likely to be low. However, the changing epidemiology of JE will require continued vigilance. Vector-control programs, the promotion of mosquito avoidance behavior, and routine immunization of children and adults in the Terai should continue. Enhanced surveillance and routine immunization of children as well as all adults should be considered.
